# Fate of Emerging Contaminants in High-Rate Activated Sludge Systems

**DOI:** 10.3390/ijerph18020400

**Published:** 2021-01-06

**Authors:** Elena Koumaki, Constantinos Noutsopoulos, Daniel Mamais, Gerasimos Fragkiskatos, Andreas Andreadakis

**Affiliations:** Sanitary Engineering Laboratory, Department of Water Resources and Environmental Engineering, School of Civil Engineering, National Technical University of Athens, 5 Iroon Polytechniou, Zografou, 15780 Athens, Greece; elenakoumaki@central.ntua.gr (E.K.); mamais@central.ntua.gr (D.M.); gerasimos.fragkiskatos@gmail.com (G.F.); aedema@gmail.com (A.A.)

**Keywords:** micropollutants, endocrine disruptors, pharmaceuticals, high-rate activated sludge, sorption, biodegradation, occurrence, removal

## Abstract

High-rate activated sludge (HRAS) systems are designed to shift the energy-intensive processes to energy-saving and sustainable technologies for wastewater treatment. The high food-to-microorganism (F/M) ratios and low solid retention times (SRTs) and hydraulic retention times (HRTs) applied in HRAS systems result in the maximization of organic matter diversion to the sludge which can produce large amounts of biogas during anaerobic digestion, thus moving toward energy-neutral (or positive) treatment processes. However, in addition to the energy optimization, the removal of emerging contaminants (ECs) is the new challenge in wastewater treatment. In the context of this study, the removal efficiencies and the fates of selected ECs (three endocrine disruptors (endocrine disrupting chemicals (EDCs))—nonylphenol, bisphenol A and triclosan, and four pharmaceuticals (PhACs)—ibuprofen, naproxen, diclofenac and ketoprofen) in HRAS systems have been studied. According to the results, EDCs occurred in raw wastewater and secondary sludge at higher concentrations compared to PhACs. In HRAS operating schemes, all compounds were poorly (<40%) to moderately (<60%) removed. Regarding removal mechanisms, biotransformation was found to be the dominant process for PhACs, while for EDCs sorption onto sludge is the most significant removal mechanism affecting their fates and their presence in excess sludge.

## 1. Introduction

Until recently, scientific interest in wastewater treatment focused on the impact and removal of conventional pollutants, especially in terms of macropollutants (i.e., suspended solids, organic carbon, nitrogen, phosphorus). The removal of these pollutants has been based on well-known biochemical reactions, such as aerobic biodegradation of organic carbon by heterotrophic bacteria, nitrification/denitrification for nitrogen removal through the combination of heterotrophic bacteria (for denitrification) and nitrifiers (for nitrification) and dephosphatation for phosphorus removal by phosphorus-accumulating organisms which can absorb dissolved phosphorus from wastewater and store it in granules within their cells. During nitrification, large amounts of dissolved oxygen are used to oxidize ammonia into nitrate (comparable to the ones required for organic carbon oxidation) and then, during denitrification, nitrate is converted through a heterotrophic process taking place under anoxic conditions into nitrogen gas and therefore consumes a significant portion of the organic matter contained in wastewater. Oxygen is transferred into the bioreactor through the aeration system, which is the primary energy consumer in treatment facilities, accounting from 50% to 70% of the energy expenditure of the wastewater treatment plant (WWTP) [1] and results in a typical energy consumption between 0.3 and 0.6 kWh per m^3^ of raw wastewater [2,3,4]. Nowadays, energy-saving and the valorization of products recovered from wastewater have become central aspects of the wastewater treatment industry [5,6]. The interest is being focused not only on the water reuse but also on the reduction in energy and carbon footprint [7].

Biogas production through anaerobic digestion of activated sludge is carried out by means of recovering the chemical energy stored in wastewater [8]; however, the low organic matter diverted to the sludge line (due to its consumption during denitrification) restricts the energy production. Recently, new configurations of wastewater treatment have been proposed that could result in up to a 60% reduction in energy consumption for aeration needs [9]. Among these technologies, High-Rate Activated Sludge (HRAS) systems are increasingly being studied [3,10]. The HRAS process relies on the high food-to-microorganism (F/M) ratios and low solid retention times (SRTs) and hydraulic retention times (HRTs), resulting in the minimization of organic matter mineralization and the maximization of its diversion to the sludge via sorption and intracellular storage as opposed to conventional activated sludge systems (CASs) [11]. The excess sludge, which is rich in organic material, can produce large amounts of biogas during anaerobic digestion [12]. The typical HRT applied is less than 4 h (usually lower than 1 h), SRT between 0.2 and 1 days, dissolved oxygen (DO) concentration below 1 mg/L and F/M ratio ranging from 2 to 10 kg BOD/kg VSS/day [13,14].

HRAS systems can be designed and optimized to meet quality standards regarding chemical oxygen demand (COD) and total suspended solids (TSS) [14], whereas ammonia and phosphorus removal efficiency is low, making a further treatment step necessary prior to the disposal of treated wastewater into nutrient-sensitive areas.

Apart from the optimization of the energy balance and the removal of conventional pollutants, a relatively new challenge of WWTPs is the removal of a wide range of chemicals—the so-called emerging contaminants (ECs). Conventional WWTPs are, in principle, not designed to remove ECs and they are only partially effective in EC removal or degradation, thus resulting in their accumulation in the receiving water bodies [15]. The occurrence of ECs in the treated effluents, the aquatic environment and the reclaimed water has become a worldwide issue receiving increasing attention, since they are characterized as being persistent in the environment, toxic and bioaccumulative and may have a substantial effect on both public health and the environment [16,17,18]. Over the last decade, different classes of ECs have been widely detected in treated wastewater, drinking water, surface water bodies, groundwater, soil and sludge [19,20,21,22,23]. ECs enter the aquatic and soil environments through diversified sources including domestic and industrial wastewater, hospital, livestock farming and agricultural activities [24]. WWTPs are considered as one of the main routes for the introduction of ECs into the environment, via either treated wastewater disposal or by the disposal or reuse of the sewage sludge in landfills and agriculture [24,25,26]. Among ECs, endocrine disrupting chemicals (EDCs) contained in everyday use products and pharmaceuticals (PhACs) receive significant research interest due to their wide use [27], their persistent occurrence in aquatic and soil matrices [20,28,29] and their estrogenic activity [30,31,32].

So far, the removal efficiency and the fate of ECs during the CAS process have been well-documented [24,26,29,33], while only one study, performed in a lab-scale reactor, is available in the literature regarding the efficiency of HRAS systems [9]. To the best of our knowledge, this study is the first of its kind, since it has been conducted in two lab-scale HRAS systems which were fed with actual municipal wastewater and the targeted ECs were at concentrations naturally occurring in municipal wastewater. The objectives of this study were to evaluate the efficiency of HRAS systems in removing the selected ECs, as well as to investigate the role of sorption and biotransformation in their removal.

## 2. Materials and Methods

### 2.1. Chemicals and Reagents

The target compounds selected for the present study were four PhACs belonging to the class of nonsteroidal anti-inflammatory drugs (NSAIDs) and three EDCs—i.e., diclofenac (DCF), ibuprofen (IBF), ketoprofen (KFN), naproxen (NPX), triclosan (TCS), bisphenol A (BPA) and nonylphenol (NP). The target compounds were selected for four reasons: (i) their widespread occurrence in raw and treated wastewater; (ii) their persistent occurrence in aquatic matrices; (iii) their high toxicity for aquatic species and (iv) the availability of data regarding their partitioning in the particulate and aqueous phases [34]. Table 1 summarizes the main physicochemical characteristics of the target compounds.

Methanol (MeOH) and ethyl acetate were of high-performance liquid chromatography (HPLC) grade (Merck, Darmstadt, Germany) and were used as received. Bis(trimethylsilyl) trifluoroacetamide (BSTFA) + 1% trimethylchlorosilane (TMCS) and pyridine, used for silylation, were purchased by Supelco (Bellefonte, PA, USA) and Carlo Erba-SDS (Peypin, France), respectively. Analytical standards of NP, BPA, TCS, IBF, NPX, KTP, DCF, deuterated BPA (BPA-d16, internal standard) and meclofenamic acid (MCF, internal standard) were supplied by Sigma-Aldrich (St. Louis, MO, USA). All compounds were used without further purification (minimum purity > 98%). Stock solutions of individual compounds were prepared in methanol at 1000 mg·L^−1^ and kept at −18 °C [35]. Stock solutions were used to prepare working standard solutions that were used for calibration. HPLC grade water was prepared in the laboratory using a MilliQ/Milli-RO Millipore system (Millipore, Billerica, MA, USA). Ultrapure HCl (32%) was used for acidification of the samples (Merck, Germany).

**Table 1:** Chemical properties of target compounds [36,37].

	EDCs			PhACs			
	NP	TCS	BPA	IBF	NPX	DCF	KFN
Structure	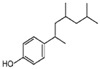	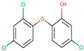	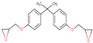	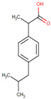	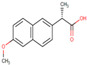	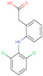	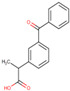
Chem. Formula	C_15_H_24_O	C_12_H_7_Cl_3_O_2_	C_15_H_16_O_2_	C_13_H_18_O_2_	C_14_H_14_O_3_	C_14_Η_11_Cl_2_ΝΟ_2_	C_16_H_14_O_3_
Mol. Weight (g/mol)	220.3505	289.5418	228.2863	206.2808	230.2592	296.1486	254.2896
Water Solubility (mg/L at 25 °C)	4.9–6.4	10	300	21	15.90	2.37	51
pKa	10.25	7.90	9.60	4.91–5.3	4.15	4.15	4.45
log Kow	5.76	4.76	3.32	3.97	3.18	4.51	3.12
log Koc	4–4.7	3.38–4.2	2.90	2.20	2.52	2.39	0.20
Henry’s Law constant (atm-cu m/mol at 25 °C)	5.6 × 10^−6^	2.1 × 10^−8^	4.0 × 10^−11^	1.4 × 10^−7^	3.4 × 10^−10^	4.73 × 10^−12^	2.1 × 10^−11^

### 2.2. HRAS Units Description and Sampling Strategy

**Figure 1:**
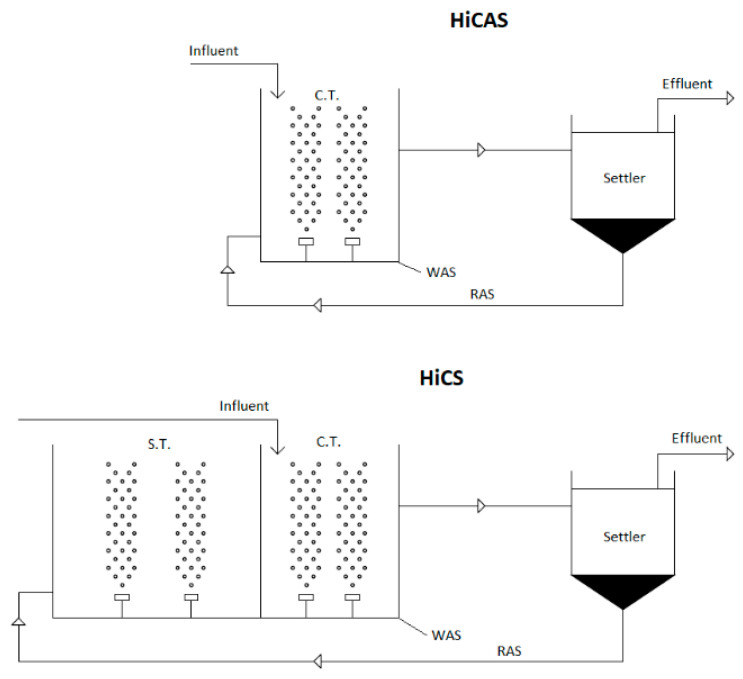
High-rate activated sludge (HRAS) lab-scale units’ layout (CT: contact tank, S.T.: stabilization tank, WAS: Excess sludge, RAS: Return Activated Sludge).

Two HRAS continuous lab-scale systems were installed and demonstrated at Psyttalia WWTP in Athens (Greece) that serves a population equivalent to 3,700,000. The two HRAS lab-scale systems’ layouts are presented in Figure 1 and the corresponding operational characteristics of the two systems are presented in Table 2. One system operated as a conventional HRAS unit (HiCAS), while the second configuration (HiCS) operated as a contact-stabilization unit. The HiCAS system consisted of a contact tank (CT), where all biological processes occurred, and a sedimentation tank to separate solids from treated water. The HiCS system is a high-rate contact-stabilization system and differs from HiCAS in that the recirculated sludge from the bottom of the sedimentation tank passes through a stabilization tank (ST) under aerobic conditions in order to create famine conditions and to enhance its ability to remove organic substances in the contact tank through improving bioflocculation, settleability, and thus carbon harvesting [38]. Both HRAS systems were fed with actual raw urban wastewater from the feed tank of Psyttalia WWTP. The quality characteristics of the raw wastewater are presented in Table 2. The volume of each contact tank (CT) was 15 L and the volume of stabilization tank (ST) of HiCS was 22.5 L. The SRTs applied in HiCAS and HiCS were 0.25 and 1.1 day, while the systems were operated at HRTs of 30 and 35 min, respectively. SRT was controlled by wasting excess sludge from the CT of each unit scheme. The dissolved oxygen (DO) in both bioreactors was maintained above 2 mg/L—hence, fully aerobic conditions were established.

COD removal efficiencies of HiCAS and HiCS were equal to 68% ± 8% and 90% ± 4%, respectively, resulting in an overload of organics in the effluent in the former case. TSS effluent concentration was 90 ± 10 mg/L for the HiCAS and 45 ± 8 mg/L for HiCS system. The lower COD and TSS removal efficiencies of the HiCAS, in comparison with the HiCS system, are attributed to the higher F/M that was applied (Table 2). Biological removal of nitrogen and phosphorus is expected to be limited and mainly attributed to the synthesis of new biomass. The low SRTs applied in both HRAS systems led to the inhibition of biological processes such as nitrification and biological phosphorus removal, thus a further polishing step is often required to meet the stringent discharge standards for nutrient removal.

Twenty-four-hour flow-proportional composite samples of influents and effluents sewage, as well as grab samples of sewage sludge, were collected during seven days within two weeks in May 2020. Wastewater and sludge samples were collected and stored in 1 L precleaned glass bottles and kept in dark. Within an hour after sampling, wastewater samples were filtered under suction through 0.45 μm membranes and stored at −18 °C for no longer than 1 day until solid-phase extraction was conducted. Membranes were stored at −18 °C for the determination of target compounds in the particulate phase until analysis.

### 2.3. Sample Preparation and Analytical Determination

For the determination of the target compounds, wastewater and sludge samples were analyzed using a chromatographic method developed by Samaras et al. [39]. The developed procedure included solid phase extraction (SPE) for wastewater samples and sonication, followed by SPE, for sludge samples. After SPE, silylation was performed at 70 °C for 20 min by adding 50 μL of BSTFA + 1% TMCS along with 10 μL of pyridine. BPA-d16 and MCF were used as surrogates for the determination of endocrine disruptors and pharmaceuticals, respectively. For the qualitative and quantitative analyses, an Agilent Gas Chromatograph 7890A connected to an Agilent 5975C Mass Selective Detector (MSD) was used. The separation of the target compounds was achieved using a HP5MS Ultra Inert GC column (30 m) with a film thickness of 0.25 μm and internal diameter of 0.25 mm (Agilent Technologies, CA, USA). Wastewater characteristics were determined using standard methods [40].

### 2.4. Data Calculation

The total concentration of the target compounds was calculated in the influent, effluent and mixed liquor samples according to Equation (1).

(1) $$C_{total}=C_{dissolved}+C_{solid}\cdot TSS$$

where *C_total_* is the total concentration of each compound (ng/L), *C_dissolved_* is the concentration of compound presented in liquid phase (ng/L), *C_solid_* is the concentration of the compound presented in solid phase (ng/g) and *TSS* is the suspended solids concentration (g/L) of the sample.

The removal efficiency of the target compounds during the treatment process was calculated from the mass flows of each compound as shown in Equation (2).

(2) $$\mathrm{Removal}\;\mathrm{Efficiency}\;\left(\%\right)=\frac{M_{infl}-M_{effl}}{M_{infl}}\cdot 100$$

where *M_infl_* is the mass of each compound in raw sewage and *M_effl_* is the mass in the effluent.

The mass balances of the target compounds in the biological unit were calculated as shown in Equations (3) and (4).

(3) $$W_{bio}=W_{infl}-W_{effl}-W_{sorb}\;$$

(4) $$W_{bio}=(Q_{infl}\cdot C_{total,infl})-\left(Q_{infl}-Q_{W}\right)\cdot C_{total,effl}-\left(Q_{W}\cdot TSS\;\cdot C_{solid}\right)\;$$

where *W_infl_*, *W_effl_* and *W_sorb_* represent mass flows of the target compounds (ng/day) corresponding to the influent and effluent sorbed onto solids removed with excess sludge, respectively. *W_bio_* is the mass flow that was lost due to the biotransformation process (ng/day). *Q_infl_* and *Q_W_* are the flow rates (L/day) of influent wastewater and waste sludge, respectively. The solid–water distribution coefficients in the sludge (*Kd*, in L/gSS) were estimated using Equation (5).

(5) $$Kd=\frac{C_{solid}}{C_{dissolved}}$$

## 3. Results and Discussion

### 3.1. Emerging Contaminants in Influent Wastewater Samples

Influent and effluent wastewater samples were collected from both HRAS systems during the sampling campaign and the data obtained are presented in Table 3. Regarding the raw wastewater, among EDCs and PhACs, the lowest mean concentrations were observed for TCS and KFN (866 ± 164 ng/L and 822 ± 336 ng/L, respectively), while the highest concentrations were observed for NP and IBF (10,896 ± 1051 ng/L and 5085 ± 1247 ng/L, respectively). For most of the PhACs, the influent concentrations detected in the present study fall into the range reported in earlier studies conducted at the same WWTP [41,42]. The only exception was the IBF, whose concentration in our study (5085 ± 1247 ng/L) was much higher compared to those previously reported, indicating IBF’s rather nonuniform use and its increasing presence in urban wastewater. This could be attributed to the seasonal fluctuation of this drug since it is usually used to reduce fever and to relieve aches and pain due to common cold and/or flu. The seasonality of the PhACs loads enter the WWTPs has also been demonstrated by others [43,44,45,46,47] and it appears that the highest concentrations are reported during the winter months. Tran et al. [29] found, from worldwide monitoring data, that IBF and NPX appear to be the most abundant NSAIDs detected in influents while they stated that distribution tendencies in influents seem to be associated with their consumption patterns, climate conditions (wet, dry, winter or summer) and population size/density. Finally, the concentrations of most target compounds were much higher than those detected in the urban wastewater of other Greek WWTPs; in Lesvos island [41], in Western [48,49,50], Northern [51] and Central Greece [22,46]. Similar results were reported by Tran et al. [52], who found high levels of ECs in raw influent of WWTPs in a highly urbanized region.

**Figure 2:**
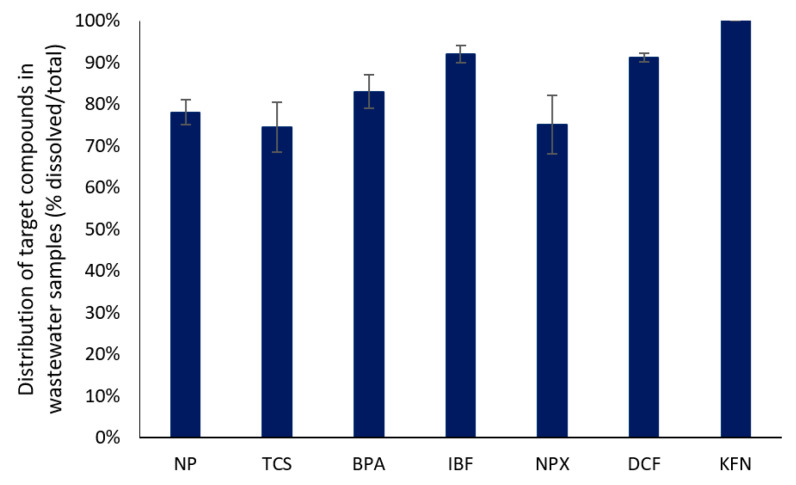
Distribution of the target compounds between dissolved and particulate phase in raw wastewater.

The distribution of the target compounds between dissolved and particulate phase in raw wastewater, as illustrated in Figure 2, indicates that the major portion of both EDCs and PhACs is in the dissolved phase. It is well-known that an organic compound with higher logK_ow_ and logK_oc_ values should normally be expected to present a higher adsorption affinity to suspended solids compared with another organic compound exhibiting a lower logK_ow_ and logK_oc_ values [53]. Therefore, the particulate portion of the target compounds is higher for NP and TCS which present high logK_ow_ and logK_oc_ values and minimal for the polar compounds (PhACs) [54]. However, this is not the case for DCF where despite its high logK_ow_, its affinity for sorption onto the solids is rather low. On the contrary, for NPX, an important part (24% ± 4%) was found in the particulate phase. Sorption capacity of these compounds onto particulates is mainly attributed to electrostatic interactions.

### 3.2. Emerging Contaminants in Sewage Sludge

Secondary sludge samples were collected from the two HRAS systems and all the target compounds, except from KFN, were detected (Table 3). The highest mean concentrations were observed for NP (3293 ± 314 and 6297 ± 2000 ng/gSS for HiCS and HiCAS, respectively) and BPA (1223 ± 179 and 1916 ± 883 ng/gSS for HiCS and HiCAS, respectively) while lower concentrations were determined for the other compounds, ranging between 140 (DCF) and 800 ng/gSS (NPX). The concentrations of the compounds were similar in the two HRAS systems with the only exception being NP, whose concentration in HiCAS was between 4437 and 8412 ng/g, and in HiCS between 3063 and 3651 ng/g. In the literature, data concentration for NP in the sludge from conventional activated sludge reactors (CAS) vary between ng/g to μg/g [41,42,55,56]. Stasinakis et al. [42] reported that NP in the secondary sludge was 1137 ± 757 ng/g, while much higher amounts of the compound were found in secondary sludge (mean concentration 24,900 ng/g) by González et al. [57]. Regarding TCS, the concentrations determined in the present study fall into the range reported in the literature; however, much higher levels were observed for BPA. In a Chinese WWTP, BPA concentration in the excess sludge was up to 198 ng/g [58], while in a study conducted in five WWTPs in California, BPA and TCS concentrations in the sewage sludge ranged between 53 and 196 ng/g for BPA and between 158 and 3277 ng/g for TCS, receptively [44]. The low concentrations of PhACs in the excess sludge has also been stated by others [20,44,59,60,61,62]. The mean concentrations of NPX in the current study were 767 ± 97 and 811 ± 128 ng/gSS for HiCS and HiCAS, respectively, higher than those reported in Spain for excess sludge (NPX: 33–51 ng/g SS) by Martin et al. [60,61].

The concentrations of ECs in sludge depend on their physicochemical characteristics, environmental and operational conditions and the biodegradation ability of the sludge system. Higher concentrations of the target compounds were observed in primary sludge compared to those in secondary sludge. This could be interpreted by the higher concentrations of the compounds in the influent wastewater or by the higher organic matter content of primary sludge [42].

It is worth noting that sorption is a phase changing process where compounds move from the dissolved phase to the solid phase (sludge and or biosolids). Therefore, it can only provide temporary risk reduction. The sorption process requires further investigations into the removal mechanism of compounds since it is yet unclear if sorption is followed by degradation or vice versa [24]. Based on the results, it can be considered that sewage sludge can serve as a major sink of the compounds with high sorption tendencies, stressing the importance of sludge management strategies. The sorbed compounds in sludge can leach out during either sludge treatment or disposal, hence necessitating a careful sludge disposal strategy. For instance, the use of sludge for irrigation activities may result in the continuous exposure of the agricultural environment to a variety of ECs, such as antibiotics, which can provide selective pressure for the development of antibiotic resistance genes (ARGs) and antibiotic resistant bacteria (ARB) [63].

### 3.3. Removal of Emerging Contaminants in HRAS Systems

The removal efficiency during HRAS treatment (Table 3) was determined using Equation (2). The significance of the difference between mass flows in the influent and the effluent of the two HRAS systems was checked using pairwise *t*-test (significance level *p* < 0.05). All compounds showed statistically significant removal rates (*p* ≤ 0.05) after treatment. However, for all compounds, except NP (*p* = 0.02), the difference in the removal efficiencies for the two operating schemes was statistically insignificant (*p* ≥ 0.05), indicating that the tested operational conditions did not affect the fate of the target compounds. In general, the removal of ECs through the different wastewater treatment processes depends on the influent mass flow of the compounds, the physicochemical properties of compounds (size, concentration, functional group, charge, polarity) and the operating conditions (SRT, HRT, pH, temperature, and the alternation of the redox conditions).

According to the results, all the compounds were poorly (<40%) to moderately (<60%) removed in both HRAS treatment systems. Specifically, PhACs presented removal ranging from 39% to 53% and from 44% to 57%, while EDC removal ranged from 48% to 62% and from 35% to 47% for HiCS and HiCAS, respectively. The total concentration of NP in the effluent of HiCS and HiCAS systems decreased from 10,896 ± 1051 ng/L, in the influent, to 5685 ± 1093 ng/L (48% removal efficiency) and to 7103 ± 1226 ng/L (35% removal efficiency), respectively. The slightly higher removal observed in the case of HiCS could be attributed to the higher SRT of the system (1.1 d for HiCS) compared to that of HiCAS (0.23 day). Higher SRT enables the incubation of slower growing microorganisms and promotes biodiversity in microorganisms with broader physiological capabilities for the enhanced biodegradation of a wider number of compounds, whereas, also, a higher HRT facilitates improved degradation of recalcitrant compounds by allowing a higher reaction time.

There is lack of studies in the literature assessing the fate of ECs and the efficiency of HRAS systems in removing them from municipal wastewater. To the best of our knowledge, this is the first study that investigated the removal of both EDCs and PhACs, naturally occurring in municipal wastewater in HRAS reactors. Recently, Taboada-Santos et al. [9] studied the fate of some ECs in a 2 L laboratory HRAS system operating at an SRT of 1 d and HRT of 2 h. According to the experimental protocol, the target ECs were spiked at the influent stream, at a concentration ranging between 1 to 20 ng/L. Taboada-Santos et al. [9] found that IBF and TCS removal was high (around 80% removal for both compounds), while the removal efficiencies for NPX and DCF were close to the values found in the present study.

Although municipal WWTPs are primarily designed to mainly remove macropollutants (i.e., organic carbon, nitrogen, phosphorus), it has been observed that they can also remove several ECs. In CAS systems, the removal efficiency varies widely, from negative removal to 100%, depending on the compound and the operating conditions of the WWTP [24,29]. CAS systems present high removal capacities for most of the target compounds (mean removal efficiency > 80%) with the exception of DCF and KFN [19]. Based on the results, it appears that the HRAS system leads to lower EC removal efficiencies compared to CAS. Both processes, HRAS and CAS, rely on the capability of the activated biomass to biotransform the ECs. Biotransformation is the breakdown of such complex compounds into simpler ones through diversified enzymatic reactions and mainly occurs via metabolism and cometabolism [29,64]. Specifically, in metabolism, the microorganisms use the ECs as substrates for cell growth and proliferation and enzyme synthesis, while in co-metabolism microorganisms rely on a primary carbon source to maintain the growth and enzyme synthesis for the EC degradation [65]. In a review article, Tran et al. [52] stated that biodegradation of ECs is mainly attributed to cometabolic activities of both heterotrophic bacteria and autotrophic ammonia oxidizers, including ammonia oxidizing bacteria (AOB) and ammonia oxidizing archaea (AOA).

In HRAS schemes, the dissolved components are eliminated by intracellular storage, biosynthesis and/or biological oxidation and the particulate and colloidal organic matter is removed from influent wastewater by biological flocculation followed by settling. Consequently, on one hand, in HRAS systems, the lower removal of the ECs could be attributed to the low HRT applied resulting in shorter reaction time that kinetically limits the biotransformation. On the other hand, it is expected that, since less COD is metabolized in HRAS systems than in CAS, this may lead to the reduction in cometabolism activity which appears to be the main biotransformation mechanism for ECs. Finally, in such low SRTs, the autotrophic ammonia-oxidizing communities are washed out leading to the limitation of nitrification, which, as demonstrated in several studies, is a key process in cometabolizing ECs [29,66,67].

### 3.4. Fate of Emerging Contaminants during HRAS Treatment

Based on the results for EDCs and PhACs occurrence in the dissolved and particulate phase and using Equation (5), Kd values, indicating the relative amount of the compounds sorbed onto particulates, were calculated for the target compounds (Table 4). The significance of the difference between the amount of the target compounds sorbed onto the secondary sludge of the two HRAS systems was assessed using a pairwise *t*-test (significance level *p* < 0.05). For all compounds, except NP (*p* = 0.007), the difference in the Kd values for the two configurations was statistically insignificant (*p* ≥ 0.05), indicating that the compounds sorbed similarly onto the sludge of the two systems. According to the calculations, EDCs presented higher Kd values compared to PhACs, with the only exception of NPX which Kd values were estimated at 1485 ± 465 and 952 ± 196 L/kgSS for HiCAS and HiCS secondary sludge, respectively. On the contrary, Kd values of PhACs were lower, not exceeding 400 L/kgSS. For compounds with Kd value less than 300–500 L/kgSS, their sorption onto sludge is considered to be insignificant [68].

Kd values calculated in the present study for IBF, DCF and KFN fall into the range reported by others in excess sludge from CAS systems [34]; whereas much lower Kd values were reported for NPX [69]. Regarding NP, TCS and BPA, similar Kd values have also been reported in previous studies [42,70,71]. On the contrary, recently, Taboada-Santos et al. [9], in secondary sludge samples from an HRAS system, found lower Kd values for IBF, NPX and DCF (16 ± 16, 18 ± 12, 21 ± 2 L/kgSS, respectively) and much higher values calculated for TCS (8748 ± 1635 L/kgSS). There are several studies where Kd values varied over a wide range [20]. The variation in Kd values of a compound among different studies could be attributed to the differences in methodology for Kd estimation. On one hand, sorption studies performed under laboratory conditions are usually conducted at higher concentrations than those found in raw wastewater, ranging from few μg/L to several mg/L; on the other hand, studies may use single point calculations rather than sorption isotherms for determining Kd. By comparing Kd values determined through lab-based experiments and field-based monitoring at WWTPs, it can be seen that the former are often substantially higher than the latter by 1 to 3 orders of magnitude [29].

Sorption mechanism encompasses both the processes of absorption onto the lipid fraction of the sludge through hydrophobic interactions and adsorption on to the surface of sludge, mostly via electrostatic interactions [72]. Specifically, the sorption is largely dependent on the physicochemical properties (i.e., logK_ow_, pKa, and ionization state) and molecular structure of the compounds, the characteristics of the sludge (i.e., biomass structure and particle size), and the operating conditions of WWTPs such as temperature, pH, HRT, SRT, redox conditions and reactors configuration, since the Kd values are governed by these parameters [29,69,71,73]. With regard to physicochemical characteristics of compounds, ECs with logK_ow_ > 4.0 or logK_oc_ > 3.0 tend to have high sorption potentials onto the particulate phase (i.e., Kd > 1000 L/kgSS), compounds with logK_ow_ < 2.5 or logK_oc_ < 1.0 often exhibit low sorption potentials (Kd < 300 L/kgSS), while ECs with logK_ow_ between 2.5 and 4 or logK_oc_ < 3.0 are more likely to have medium sorption potentials [19,53]. For instance, TCS shows hydrophobicity based on its characteristics under environmental pH 6–8 (pKa: 7.9, logK_ow_: 4.76 and logK_oc_: 3.38–4.2) (Table 1); hence TCS’s Kd values are expected to be calculated higher than 1000 L/kgSS. Moreover, pKa value is crucial parameter when charge interactions play a role in sorption. Specifically, at a pH higher than pKa, the ECs that contain hydroxyl groups may be negatively charged and thereby their sorption will be hinder through charge repulsion. PhACs present as neutral and anionic species (pKa < 5) in wastewater pH values, usually around 7, and have low logK_ow_ values. Therefore, their sorption onto sludge is expected to be weak due to the electrostatic repulsion from the negatively charged functional groups in the activated sludge and consequently the detected concentrations are low (Table 3). 

The main mechanisms taking place in activated sludge systems that contribute to the removal of ECs are biotransformation and sorption, whereas other mechanisms such as photodegradation, volatilization and hydrolysis have minimal impacts on the fate of ECs. Photodegradation during biological treatment is insignificant due to the sunlight blockage by highly concentrated particulate matters present in wastewater. According to the physiochemical properties of the target compounds (Henry’s law constant), it seems that the target compounds are not volatile and, therefore, volatilization during secondary treatment is not expected to be an important process. Hydrolysis is also not expected to occur due to their chemical structure—they lack functional groups that can be hydrolyzed under environmental conditions (pH 5 to 9) [74].

**Figure 3:**
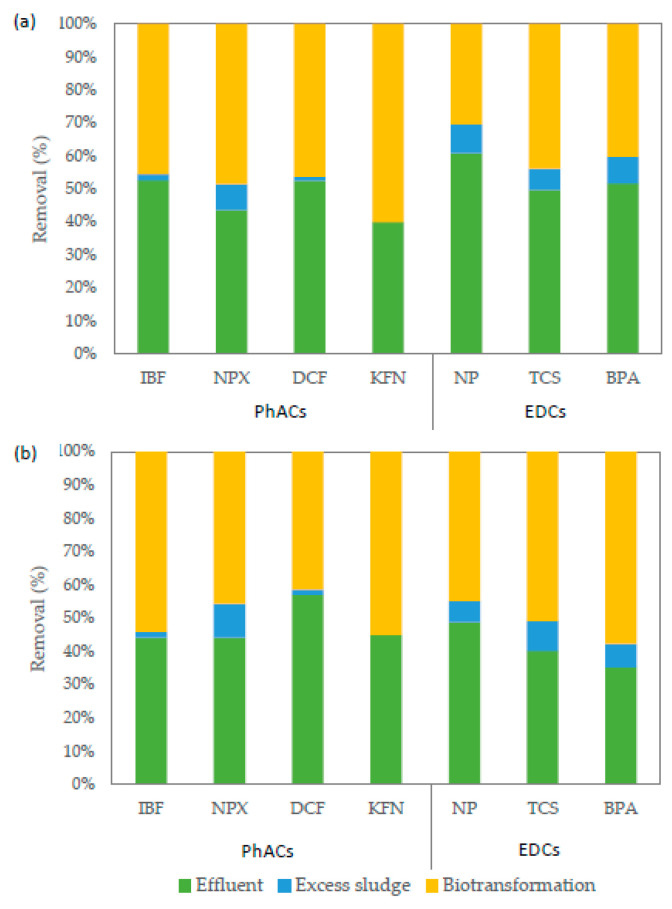
Contribution of sorption and biotransformation to the overall percentage removal of the target compounds in (**a**) HiCAS and (**b**) HiCS system.

Figure 3 presents and compares the removal efficiencies of the target compounds achieved in the two HRAS systems. According to the results shown in Figure 3, all compounds are mainly removed from the system via biotransformation. The mean removal due to biotransformation estimated for NSAIDs ranged from 46% (IBF and DCF) to 60% (KFN) and from 41% (DCF) to 55% (KFN) in HiCAS and HiCS systems, respectively. Lower biotransformation was observed for EDCs ranging from 30% (NP) to 44% (TCS) and from 45% (NP) to 58% (BPA) in HiCAS and HiCS systems, respectively. Regarding EDCs and NPX, an important component seems to be carried away from the system through the removal of excess sludge. Specifically, NP, TCS, BPA and NPX are removed from HiCAS via sorption onto secondary sludge at 22%, 13%, 17% and 14%, respectively. For IBF, DCF and KFN, it was estimated that less than 2% of the initial mass was removed via sorption and the major amount of the substances was biotransformed in both systems (Figure 3). Taboata-Santos et al. [9] reported similar removal efficiencies for DCF (10–20%) and NPX (40–70%) in a HRAS system, whereas higher removal efficiency was observed for TCS and IBF (around 80%). Moreover, in the cases of IBF, DCF and NPX, they found that the entire removal took place via biotransformation, while for TCS, half of its removal was attributed to sorption.

Low HRT limits the contact time between microorganisms and compounds and low SRT restricts the selection of organisms that are able to transform these compounds. Gros et al. [75] linked biodegradation rates (Kbiol) with HRT and found that compounds with high Kbiol and low Kd values are more influenced by HRT, while HRT does not affect compounds with low Kbiol and low Kd values. Moreover, they found that ECs with low Kbiol and high Kd values are more influenced by SRT due to the impact of the sorption. The target compounds based on their Kbiol values are categorized as follows: IBF and BPA appeared to be easily biodegraded during biological wastewater treatment processes since their removal efficiencies are often greater than 90% (Kbiol > 10 L/gSS d); NPX, DCF, KFN, NP and TCS appeared to be moderately removed by biodegradation processes and their efficiencies ranged from 20% to 90% (0.1 < Kbiol < 10 L/gSS d) [22,24,34]. Based on the literature data for Kbiol values of the compounds, it can be considered that, in HRAS systems, most of the compound biotransformations are limited by the low HRT applied and by the kinetic limitations that occur at higher loading rates of the ECs.

Studies performed in CAS systems have also shown that physicochemical processes, such as sedimentation and precipitation, resulting in EDC removal from the dissolved phase through the sorption onto primary and secondary sludge [55,56], while for PhACs, sorption onto sludge is not considered as a significant removal process [76]. Therefore, biodegradation seems to be the dominant mechanism for the removal of acidic PhACs from CAS systems [66,76,77]. Nevertheless, significant biological removal rates have also been observed for EDCs [78]. Clara et al. [79], in CAS laboratory-scale experiments, observed 90% reduction in BPA and IBF concentrations at SRTs of 13 and 10 d, respectively, but when SRT decreased to 2 d, the removal was negligible (<20%), indicating the role of nitrification activity. A positive correlation between the microorganism’s concentration and IBF removal has also been reported by Collado et al. [77] in experiments performed with biomass from a CAS reactor. Regarding NP, it seems that although biodegradation is a crucial process in CAS systems, and at the same time a significant amount of its mass is sorbed onto the sludge [41,55,56]. The poor removal of DCF (21–40%) observed in CAS systems, mainly due to low biodegradation, has been reported by Zhang et al. [80]. Finally, with regard to NPX, although studies carried out in WWTPs have shown that its main removal mechanism is biodegradation, the removal efficiency may vary widely depending on the operational parameters of the facilities (HRT and SRT) and the seasonal fluctuations that may change the input loads. Specifically, a study conducted by Metcalfe et al. [81] showed that for HRT longer than 12 h, the removal of NPX was enhanced, while also the biodegradation increased at a higher SRT [34].

Based on the findings from the present work, it can be stated that the establishment of HRAS treatment schemes for optimizing the energy footprint of WWTPs, which tend to favor low SRTs and HRTs, appear to be at odds with strategies to enhance biotransformation of ECs. HRAS biological treatment processes are unable to satisfactorily remove persistent and/or moderately biotransformed ECs; however, efficiency can be improved through a multibarrier approach where advanced tertiary treatment processes could be employed following the HRAS treatment process. Further evaluations of the biodegradation of ECs and sorption in HRAS processes would be beneficial while further advancements in HRAS technologies are necessary for the efficient removal of ECs (improvement and optimization of the operating conditions).

## 4. Conclusions

The removal efficiencies and the fates of selected EDCs and PhACs have been studied in HRAS systems treating municipal wastewater from a heavily urbanized region. The target compounds occurred in different concentration levels in the dissolved and particulate phases and presented different fates during a HRAS treatment process. In raw wastewater, among EDCs and PhACs, the highest mean concentrations were observed for NP and IBF and the lowest concentrations were observed for TCS and KFN, respectively. Similarly, in secondary sludge the highest mean concentrations were observed for NP and BPA, while lower concentrations were determined for the other compounds. Compared to CAS systems, HRAS biological treatment processes appear to achieve lower overall removals for the selected ECs studied. In both HRAS operating schemes, all compounds were poorly (<40%) to moderately (<60%) removed, indicating that the tested operational conditions did not significantly enhance the removal of target compounds. Particularly, EDCs were removed by 35–62%, while PhACs removal ranged between 39% and 57% in both HRAS configurations. In general, PhACs presented lower distribution coefficient values (Kd) in secondary sludge compared to EDCs, indicating that their sorption onto sludge is insignificant and biotransformation is the dominant removal mechanism in HRAS schemes. On the other hand, in the case of EDCs, both biotransformation and sorption govern their fates in HRAS systems.

## Figures and Tables

**Table 2:** Operational parameters of the two HRAS systems.

	HiCAS	HiCS
Influent flowrate (L/day)	720	580
Volume (L)	15	15/22.2 ^1^
Excess sludge (WAS) (L/day)	48	38
Return Activated Sludge (RAS) %	100	150
HRT (h)	0.5	0.625/0.625 ^1^
SRT (days)	0.25	1.1
DO (mgO_2_/L)	>2	>2
T (°C)	20 ± 2	20 ± 2
MLSS (g/L)	2.3 ± 0.24	3.2 ± 0.15/4.9 ± 0.35 ^1^
F/M (kgCOD/kgVSS/day)	14	9.5
tCODin (mg/L)	675 ± 50	675 ± 50
TSSeff (mg/L)	90 ± 10	45 ± 8
tCODeff (mg/L)	220 ± 50	70 ± 20
tCOD removal (%)	68 ± 8	90 ± 4

^1^ (contact tank)/(stabilization tank).

**Table 3:** Removal efficiency (%) and occurrence (average ± standard deviation) of the target compounds in influent and effluent wastewater (dissolved and particulate phase) and in secondary sludge of the two HRAS systems.

	Compound	Influent (ng/L)	HiCAS			HiCS			
			Effluent (ng/L)	Sludge (ng/gSS)	Removal Efficiency (%)	Effluent (ng/L)	Stabilization Tank (ng/L)	Sludge (ng/gSS)	Removal Efficiency (%)
EDCs	NP	10,896 ± 1051	7103 ± 1226	6297 ± 2000	35% ± 9%	5685 ± 1093	21,860 ± 919	3293 ± 314	48% ± 8%
	TCS	886 ± 164	473 ± 232	373 ± 106	47% ± 19%	381 ± 202	2345 ± 221	379 ± 35	57% ± 18%
	BPA	3578 ± 479	1983 ± 856	1916 ± 883	45% ± 25%	1347 ± 652	7962 ± 1739	1223 ± 179	62% ± 21%
PhACs	IBF	5085 ± 1247	2869 ± 665	607 ± 175	44% ± 17%	2405 ± 512	4474 ± 657	552 ± 198	53% ± 22%
	NPX	1572 ± 483	735 ± 237	811 ± 128	53% ± 10%	743 ± 136	4498 ± 432	767 ± 97	53% ± 20%
	DCF	1920 ± 432	1081 ± 223	142 ± 91	44% ± 14%	1171 ± 165	1890 ± 135	149 ± 92	39% ± 18%
	KFN	822 ± 336	353 ± 61	<LOD ^1^	57% ± 19%	396 ± 34	385 ± 19	<LOD	52% ± 23%

^1^ LOD: Limit of detection.

**Table 4:** Distribution coefficient values (average ± standard deviation), Kd (L/kgSS) for the target compounds in secondary sludge of the two HRAS systems.

	Compound	HiCAS	HiCS
EDCs	NP	1081 ± 116	669 ± 85
	TCS	1260 ± 843	1322 ± 596
	BPA	1409 ± 813	648 ± 115
PhACs	IBF	243 ± 97	211 ± 106
	NPX	1485 ± 465	952 ± 196
	DCF	152 ± 124	124 ± 82
	KFN	-	-

## Data Availability

Data available on request. The detailed experimental data presented in this study are available on request from the corresponding author.
